# Overcoming language barriers in paramedic care: a study protocol of the interventional trial ‘DICTUM rescue’ evaluating an app designed to improve communication between paramedics and foreign-language patients

**DOI:** 10.1186/s12913-020-05098-5

**Published:** 2020-03-18

**Authors:** Eva Maria Noack, Evelyn Kleinert, Frank Müller

**Affiliations:** Department of General Practice, University Medical Center Göttingen, Humboldtallee 38, 37073 Göttingen, Germany

**Keywords:** Paramedic care, Interpreter, Medical translation application software, App, Digital communication tool, Foreign-language patients, Language barrier

## Abstract

**Background:**

It is essential for medical treatment that patients and medical staff can communicate about acute complaints, pre-existing conditions, and the treatment procedure. Misunderstandings can have far-reaching consequences, particularly in time-critical emergencies, which require rapid assessments and decision-making and in which interpreters are rarely available.

In this study, we aim to develop a digital communication tool that is to help paramedics communicate with patients who speak hardly any or no German, to monitor its implementation, and to investigate its effect on communication between foreign-language patients and staff. Furthermore, a large amount of data on patients that are cared for in emergency medical services in Germany are collected for the first time.

**Methods:**

To consider the complex situations of paramedic care and to meet paramedics’ demands, we use an action-oriented research approach to develop the tool. We include the staff of the participating emergency medical service stations and software designers in our approach. The tool is then used and evaluated within an open interventional, non-randomised study with two control groups. Control group 1 (German-speaking patients) and control group 2 (non-German-speaking patients treated without the tool) are recruited starting from the first study phase. In the second study phase, an intervention group is additionally recruited, i.e. non-German-speaking patients with whom the tool is used.

The primary outcome of the clinical trial is improved communication with non-German-speaking patients in emergencies by means of the communication tool. The secondary outcome is an improved quality and quantity of the collected information. We exploratively observe on-scene times, demands for emergency physicians, and the usage of the intervention. By recording patients’ clinical parameters, we consider the severity of the health restrictions.

**Discussion:**

Our study is an innovative research project in paramedic healthcare comprising the development of a digital communication tool to overcome language barriers in emergency medical services and investigating its usability, acceptance, and effect on communication, in short, its usefulness and value for paramedic care. Additonally, we expect to gain comprehensive information on rescue operations.

**Trial registration:**

German Clinical Trials Register, DRKS00016719, registered 08 February 2019, World Health Organization Trial Registration Data Set, http://apps.who.int/trialsearch/Trial2.aspx?TrialID=DRKS00016719

## Background

### Language barriers in the provision of paramedic care in Germany

Almost one-fourth of Germany’s population, 20.8 million inhabitants, have a migration background [1]; 10.6 million people have a foreign citizenship [2]. Additionally, 180,000 to 520,000 undocumented migrants lived in Germany in 2014 [3]. This number has presumably increased in recent years. No official data on the language skills of migrants in Germany are available [4]. Even though the majority probably speak German fluently, a substantial number is assumed to be unable to communicate sufficiently in German. This number of migrants living in Germany with no or little knowledge of German has probably increased in recent years because about 0.9 million asylum seekers and refugees came to Germany between 2015 to 2017 [5]. In addition to foreign residents, 37.5 million foreign tourists visit Germany per year (data for 2017) [6]. Germany is the most important destination for business trips in Europe [7], and is a transit country for travellers and long-distance transports. Moreover, 286,000 seasonal agricultural workers come to Germany every year (data for 2016) [8], many of them from Eastern Europe.

Some of these non- or barely German-speaking residents, tourists, business travellers, or migrant labourers may need medical emergency care during their stay in Germany. Communication about acute symptoms and pre-existing conditions is the basis of any medical treatment. In a representative study in 2006/2007 on the largest foreign nationality groups at that time (from Turkey, former Yugoslavia, Italy, Greece, and Poland), 10% of the respondents declared that they encountered difficulties in medical consultations due to insufficient language skills – even though the very large majority of these respondents had been living in Germany for more than 8 years, most for even more than 20 years [4, 9]. The German Government estimates that about 20% of all new immigrants will permanently need interpreter assistance when using health services [10]. Research has shown that refugees and asylum seekers in Germany experience difficulties when seeking health care, especially due to language barriers. This limits physician-patient communication [11–13]. Especially when no interpreters are available, situations of mutual incomprehension are a great challenge for patients who speak hardly any or no German and the medical staff who treat them.

In emergency care, the option of on-site interpretation is mostly very limited. Migrant and refugee patients in Germany are more likely than Germans to seek paramedic and out-of-hour medical care, however [14, 15]. Especially in out-of-hospital medical emergencies it is crucial for the paramedic staff to understand the current complaints of the patients, their characteristics and duration of acute symptoms as well as pre-existing conditions, allergies, or drug treatments. Communication difficulties can lead to inaccurate initial assessment and misdiagnosis, which may increase the risk of ensuing incorrect clinical decisions and treatment. This can have potentially life-threatening consequences for the patients. It might also cause unnecessary psychological stress to both patients and medical staff.

Some findings have indicated that the quality of care is inferior for patients with limited language proficiency: Language barriers delay transport to hospitals [16], even though in many emergencies, time is a crucial factor for the patient’s outcome. Language barriers may increase the likelihood of (unnecessary) intubation and intensive care during rescue missions with trauma patients [17]. Lee et al. [18] find that language barriers between emergency physicians and adult patients increase the probability of being admitted to hospital – probably partly as a precautionary measure.

Ignorance of the medical history of the patients and/or misinterpretation of symptoms may also lead to incorrect decisions regarding the choice of hospital. Especially in rural areas, paramedics often have to decide whether to take a patient to a small hospital that is close by and provides basic medical care (or choose between different small hospitals that provide different medical specialities such as obstetrics, chest pain units, or stroke units) or to take the road (and the time) to a hospital that offers maximum medical care.

Moreover, the recent Emergency Paramedics Act (Notfallsanitätergesetz, NotSanG) in Germany has given more competence and responsibility to the paramedic staff so that they can autonomously provide therapeutic measures. Understanding the patients’ situations has therefore become even more important for therapeutic decision-making in the field.

In summary, language barriers play an important role in paramedic care in Germany. Overcoming these barriers might be of high value for patients, health care providers, and the emergency medical system. Digital communication tools designed for the use in health care may help to improve communication in medical consultations [19]. The implementation of a tablet-based application software (app) for medical history taking in a German transit camp for refugees and asylum seekers has shown that this is accepted by most patients and can be implemented well [20, 21].

However, this app was developed for patients seeking primary care and who may enter their data themselves; the information is then translated for the general practitioner. Emergencies require a different content and a different approach: Completely different clinical pictures and diseases have to be considered. The handling must be done by the medical staff, resulting in different user interface and menu navigation. Paramedics may not only be required to gather information from the patients, but also to give them essential information. Additionally, time pressure in emergencies makes it challenging to develop a reliable, rapid-to-use application software: On average, on-set time of medical emergency missions before transportation to hospital is 16.9 min with and 22.3 min without accompanying emergency physician [22].

### Medical emergency care in Germany

Medical emergency care in Germany is typically organised and provided on the district level. It is either provided directly by the municipality or district (“kommunaler Rettungsdienst”), or it is contracted to non-profit organisations or private companies. Costs incurred by the rescue service are reimbursed by the health insurance.

There are regulations regarding qualifications of paramedic and medical staff and respective personnel on different types of emergency vehicles. Some of these regulations differ from state to state, but generally, paramedics provide the first level of emergency care; emergency physicians provide backup in life-threatening situations. The emergency physician is typically a physician with special training and a board certification in emergency medicine. There are currently two non-physician emergency professions: In 2015, the “Rettungsassistent”, requiring a 2-year theoretical and practical training, was replaced by the profession of “Notfallsanitäter”, a highly professional emergency care provider. This requires 3 years of training. A Notfallsanitäter has more responsibility because they may provide therapeutical measures without prior consultations of the emergency doctor, and they have more competencies to assist the emergency physician on scene on a high level. Paramedics are thus trained to respond to emergencies and can handle most rescue missions by themselves. Emergency physicians are only called out to seriously injured or critical patients to provide intense on-scene care and typically join the paramedic staff on the emergency scene (rendez-vous approach). Fewer than one-fifth (18%) of all the emergency operations in Germany include an on-site emergency physician, however [22].

Most recent data on emergency operations in Germany are from 2012/2013: approximately 12 million operations were carried out Germany-wide by public paramedic services. The deployment rate was 147 responses per 1000 inhabitants and year. However, only approximately half of these responses were emergency operations. The other half were ambulance transport services (transport of non-emergency patients, e.g. home from hospital). The time span until paramedics arrived on the scene averaged 8.4 min. Within 16.9 min, nearly all (95%) emergency sites were serviced [22].

### The aim of the study

In this study, we aim at developing, implementing, and evaluating a digital communication tool that helps paramedics to obtain (and give) essential information from (and to) non-German-speaking patients in their native language or in a language they are proficient in, and thus to overcome language barriers. We also wish to assess how the intervention affects the quality of information gathered from patients and how the paramedic staff perceives it. For this purpose, we conduct a clinical trial. We expect to gain comprehensive understanding of the possible benefits and constraints for the use of this tool in paramedic care. In addition, comprehensive information on healthcare encounters with non-German-speaking patients in emergency medical services is collected for the first time.

## Methods and study design

**Fig. 1:**
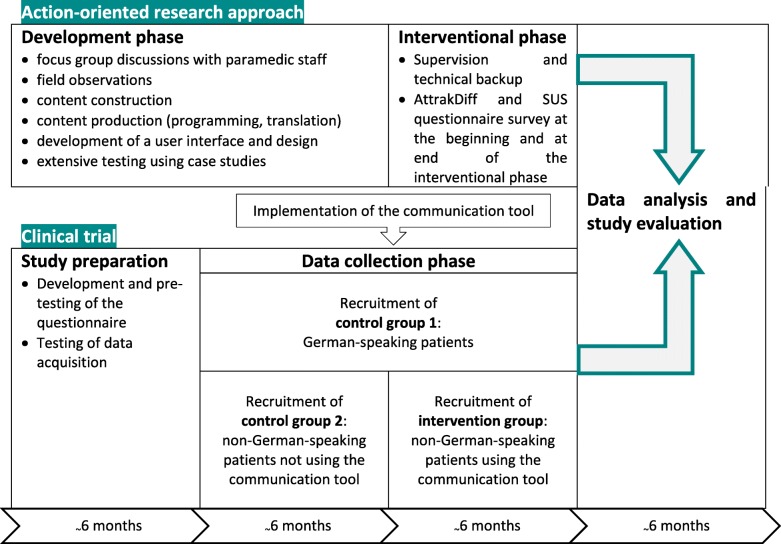
Timeline of the study

Different methodological approaches will be combined for the different study objectives of development, implementation, and evaluation. These are carried out in different time frames (cp. Fig. 1).

First, the communication tool will be developed using an action-oriented research approach within the first 12 months of the project. Simultaneously, a clinical study will be prepared and data of two control groups will be recruited. Then, the developed app will be implemented into the emergency service procedures, and the phase of recruiting the intervention group begins. Finally, we will evaluate the obtained data. In the following, the methodological approaches are described in more detail.

### Action-oriented research approach for development and implementation

#### Data collection and data analysis

The communication tool will be developed and implemented using an action-oriented research approach. This implies that researchers will develop the intervention in close collaboration with actors in the field, in this case, the paramedic staff of four emergency medical service stations in central Germany (see below, study site and study setting). Paramedics will be involved in focus group discussions, informal background discussions, case conferences, and analyses of emergency cases with non-German-speaking patients. In addition, paramedics will perform extensive testing with beta versions in role-play test cases. Focus group discussions and testings with role plays with case trainings will be documented by the researchers by creating minutes and result logs. The suggestions by the paramedics for modification will be discussed and subsequently included in the development process. This is intended to be a circular process with several rounds of discussions and adjustments.

We will use these techniques to provide input for the communication tool in a first step, and to improve the content and user interface design of the app in a second step. An advantage of this approach is that it will enable us to consider perspectives from different stakeholders, to understand and take into account the needs of end users, and to respond flexibly to unexpected challenges that arise when the app is used in the field. We will also discuss and plan the implementation of the tool into the daily routine to anticipate potential obstacles in practice. This approach therefore ensures that the tool is ready to be used in the daily routine of the paramedics.

If no significant new requests for modification are reported, we will develop a first stable version of the app, which is to be tested with patients for a period of several weeks, accompanied by field observations and supported by technical backup. Suggestions for improvement and any necessary adjustments will be introduced in a final version, which will then remain unchanged during the clinical trial phase of intervention group recruiting.

Interpretation and processing of data in this project are oriented towards the goal of developing and implementing the communication tool. We therefore use the technique of identifying central statements as proposed by Ruddat [23] to create the tool requirement profile.

In addition, quantitative questionnaire surveys will be conducted shortly after the successful implementation and after 6 months to determine the usability and the subjectively perceived quality and attractiveness of the app. To assess the usability of the app, we will use the System Usability Scale (SUS) by Bangor et al. [24], and for its perceived quality and attractiveness we will use the AttrakDiff Questionnaire by Hassenzahl et al. [25]. Additionally, we will collect socio-demographic information on the paramedics such as age, gender, professional qualifications, and work experience. These factors may affect their willingness to use a digital tool and their perception of it. This two-stage survey enables us to assess whether and how the perception and acceptance of the tool change over time.

The questionnaires on the usability, quality, and attractivity of the app will be analysed mainly using descriptive statistics. Correlation analyses will be carried out aditionally to identify, for example, associations between the demographics of the paramedics and the responses they make to the questionnaires. We will thus use quantitative methods for analysing the questionnaire surveys, and a qualitative analysis to summarise focus group discussions and testings.

#### Recruitment of paramedic staff and informed consent

Before we start the action-oriented research phase and the first data-collection phase for the clinical trial, all paramedics of the four participating emergency medical service stations will be informed about the aims and process of the study. The paramedic staff are then invited to participate in focus group discussions and testing rounds with role plays in test cases. Inclusion criteria are thus met by all paramedics who are employed in one of the four rescue stations. They need to declare informed consent for participation and for documentation of the discussion processes and results. Paramedics have the right to withdraw from the study at any point, and any data containing personal information will be deleted immediately. However, minutes and result logs will not contain any personal data, and questionnaire data are also collected anonymously. This means that any given information cannot be deleted. We expect the complete paramedic staff (*n* = 131) to take part in the study. There are 23 paramedics in Wendhausen, 28 in Königslutter, 40 in Helmstedt, and 40 in Braunschweig. Focus group discussions are planned with 6–8 paramedics. Participation will change, so that ultimately a large part of the staff of each rescue station will have been involved in the development process. Moreover, we will ensure that female and male paramedics and paramedics with different professional experience are involved in the development process.

### Clinical trial

In order to evaluate the effect of the developed app, we will conduct a prospective open intervention study. The main purpose of the clinical trial is to assess how the use of the app affects the perceived quality of understanding of non-German-speaking patients from the paramedics’ point of view **(primary outcome)** and how it affects the quality and quantity of information obtained from patients with language barriers **(secondary outcome).** In addition, we will assess whether the tool is perceived as helpful and easy to handle in daily use by the paramedics.

For this purpose, two different patient control groups are recruited and compared with one interventional patient group. Control group 1 consists of German-speaking patients, whereas control group 2 consists of non-German-speaking patients with whom the communication tool is not used. The interventional group consists of non-German-speaking patients with whom paramedics use the tool to communicate with patients. German-speaking control group 1 serves as baseline for on-scene times, intubation rates, and requests for on-scene emergency doctor support. The recruitment period for this group will be at least 12 months because a very large total number of cases is necessary for the selection of matched control groups. By comparing data on control group 2 and on the interventional group, the immediate effect of the tool on communication and information gathering with non-German-speaking patients can be analysed.

As to the primary outcome, we expect that using the tool, paramedics perceive the communication with non-German-speaking patients as easier and more fruitful. Therefore, we will analyse paramedics’ feedback on questionnaires for each emergency drive with foreign-language patients concerning the communication and mutual understanding with patients (see below). Paramedics are asked to report their experience with the tool to assess its acceptance and usability (see above).

Moreover, this study attempts to generate first knowledge about the extent and relevance of language barriers in emergency operations. With regard to the secondary outcome (quality and quantity of information obtained from the patient), we will be able to compare non-German-speaking patients with German-speaking patients and take demographic and medical aspects into account.

#### Data collection

Within the clinical trial, data from three different sources are collected and analysed. In order to be able to combine these different data sources on a case-by-case basis, the operational number of each emergency case is used as a pseudonym. This is deleted when the data sources have been matched.

- Rescue Service Case Protocol (‘DIVI-Protocol’).

It is mandatory to document all relevant medical information of any emergency operation served by paramedics in standardised Rescue Service Case Protocols called “DIVI-protocols” (**D**eutsche **I**nterdisziplinäre **V**ereinigung für **I**ntensiv- und Notfallmedizin e.V., the German Interdisciplinary Association for Intensive and Emergency Medicine). We will obtain and analyse extracted and anonymised information of these rescue service protocols of all rescue missions that occur at the four emergency medical service stations during the data collection period (listed in Table 1). These data will allow us a comprehensive overview of the characteristics of rescue operations with both German- and non-German-speaking patients. This is the first investigation of its kind in Germany. We will also compare the quantity and quality of information between patients in the intervention group and in the control groups and match these data with the questionnaires that paramedics are to fill out on emergencies with non-German-speaking patients and data on the usage of the app (see below).

**Table 1:** Data on rescue missions obtained from the DIVI-protocols

**Patient demographics**	• Year of birth • Sex
**Emergency Operation**	• Operation site (apartment, retirement home, work place, sports place, doctor’s practice, birth centre, hospital, public space, road, school, educational facility, mass event, other) • Other involved emergency vehicles (NEF*, RTW etc.) • Time of emergency call (hh-mm) • Time of arrival at emergency scene (hh-mm) • Time of arrival at patient (hh-mm) • Time of departure at emergency scene (hh-mm) • Time of arrival at hospital (hh-mm)
**Emergency Case**	• Paramedic’s description of emergency case: event, patient’s medical history, pre-medication (free text) • (Working) diagnosis (free text) • Necessity of ventilation (laryngeal mask, laryngeal tube, endotracheal intubation)
**Medical Aspects**	• Initial Glasgow Coma Scale • Initial NACA-Score • Conditions (stroke, seizure, meningitis, syncope, acute coronary syndrome, STEMI, cardiac arrhythmias, pulmonary embolism, heart failure, orthostatic misregulation, pulmonary edema, cardiogenic shock, pacemaker malfunction, asthma attack, COPD, pneumonia, bronchitis, hyperventilation syndrome, aspiration, haemoptysis, acute abdomen, gastrointestinal bleeding, colic pains, enteritis, psychotic disorders, depression, anxiety, intoxication, suicide (attempt), psychosocial crisis, hypoglycaemia, hyperglycaemia, dehydration, uraemia, fever convulsion, pseudocroup, SIDS, near-SIDS, pregnancy, impending birth, (pre)eclampsia, vaginal bleeding, anaphylactic reaction, heat stroke, hypothermia, frostbite, high fever infection, sepsis, septic infection, influenza, hepatitis / hiv, acute lumbago, epistaxis, social problem (without mental disorder), medical treatment complication) • Injuries (skull & brain, face, neck, thorax, abdomen, spine, pelvis, upper extremities, lower extremities, soft tissues)

*Abbreviations*: *NEF**N**otarzt**e**insatz**f**ahrzeug, i.e. non-transporting emergency physician rapid response car, **RTW**R**ettungs**t**ransport**w**agen, i.e. patient-transporting ambulance, *NACA* National Advisory Committee for Aeronautics, *STEMI* ST-elevation myocardial infarction, *COPD* chronic obstructive pulmonary disease, *SIDS* sudden infant death syndrome

At the emergency medical service stations in Wendhausen, Königslutter, and Helmstedt, the DIVI-protocols are filled in using a laptop device. The responsible district of Helmstedt will provide us a pseudonymised digital dataset. In Braunschweig the staff uses paper DIVI-protocols that will need to be digitalised. Paper protocols will be scanned and processed with the Optical Character Recognition software Form Pro 3.0 (OCR Systeme GmbH Leipzig, Germany), and will subsequently be reviewed to correct misrecognised values.

For the collection and use of DIVI-protocol-based research data, agreements have been reached with the local authorities of the district of Helmstedt and the City of Braunschweig on the basis of existing laws on the execution of rescue services in Lower Saxony.

b) Questionnaire on emergencies with non-German-speaking patients

The DIVI-protocol by default does not contain any information about communication and mutual understanding. We will therefore introduce a questionnaire to collect these data. Paramedics are asked to fill out questionnaires for all emergencies with patients whose native language presumably is not German. The aim is to determine how often paramedics encounter language barriers, how they experience these emergency operations, and how paramedics’ experience changes when the app is used during the interventional phase. For each rescue mission, the questionnaire can be matched with the corresponding DIVI-protocol. An ambulance is operated by at least two and up to four paramedics, therefore the person who has communicated most with the patient is to complete the questionnaire. The questionnaires should be completed immediately after the operation, but no later than 24 h thereafter. The content of the questionnaire is shown in Additional file 1.

c) Data collected from the mobile device.

Data on paramedics’ use of the app will be collected. This concerns the selected language and e.g. the order of the questions presented to the patient and different types of touch inputs, including ‘missed clicks’ or cancelling. We can then calculate, for example, the time-to-click intervals and average time for completion. This information helps us to understand how paramedics use the app, which questions they ask, and in which order and how they handle the user interface. We can compare the information from the DIVI-Protocols with the use of the app and try to assess the accuracy of the information that is collected through the app. We can also determine the affiliation to a language group of non-German-speaking patients with whom communication is difficult.

#### Recruitment and informed consent

Within the clinical trial, data are collected about two different parties. On the one hand, patients from whom medical and demographic data are collected, and on the other hand, paramedics, who express their perspective on the communication with patients, their asssessment of the app, and of whom data will be collected when they use the app. All these data will be collected anonymously. Whether paramedics use the app is exclusively their decision and responsibility. Clear rules will be put in place so that under no circumstances rescue personnel or patients are endangered because of use of the app. Using the app and participating in the study is voluntary. Paramedics need to declare written informed consent (see above).

No written consent is obtained from the patients because it can be assumed that their well-being is not endangered by the intervention. Obtaining informed consent is desirable, but under the circumstances of an emergency operation, we would meet insurmountable hurdles with obvious ethical conflicts. Adequate informed consent seems hardly possible with patients in extreme medical situations and who do not speak German. In addition, giving adequate information would prolong the time span of the rescue mission and delay transport and thereby endanger patients and paramedics alike. Obtaining an informed consent retrospectively is likewise associated with obstacles because study participants may come from abroad, and collecting contact details (telephone number, name, address) – and explaining why these are asked for – is equally difficult in an emergency situation (and some patients may not wish to give their contact details, e.g. due to their residence status). However, if a patient objects to communicating via the device, paramedics will abort the use. Only anonymised patient data are collected, and this is done in a way that effectively prevents retrospective identification.

#### Inclusion criteria for patients

Patients who meet the following criteria are eligible to participate in the study:

- Control group 1: German-speaking patients of all ages who do not use the app.
- Control group 2: Non-German-speaking patients of all ages who do not use the app (i.e. within the first period of recruitment and because of drop-out in the second recruitment stage)
- Interventional group: Non-German-speaking patients of all ages who speak one of the available languages (cp. Table 2) and who are responsive to use the app.

**Table 2:** Supported languages

• Arabic: possible dialects are Syrian Arabic, Egyptian Arabic, Moroccan Arabic (spelling Modern Standard Arabic) • Bosnian • Croatian • Czech • Dari • English • French	• Italian • Kurdish: possible dialects are Sorani and Kurmanji • Lithuanian • Pashto • Persian • Polish • Russian • Serbian • Turkish

#### Drop-out criteria for patients from the interventional group

When the intervention takes place, paramedics can refuse to use the app at any time. They are not to use the app when it presumably places the patient or themselves at risk (e.g. rescue operation on a busy and unblocked motorway). Furthermore, the app is not to be used in emergencies with immediate action implications (e.g. cardiopulmonary resuscitation or transport to the hospital necessary without any delay) or if the patient has a severe limitation of consciousness. If patients do not speak languages provided in the app (cp. Table 2), they are excluded from the interventional group but serve as control patients in group 2. Patients who meet the eligibility criteria but with whom paramedics do not use the intervention will be analysed separately.

#### Data management

Personal information allowing patient identification is neither collected by the app nor transferred in the extract of the DIVI-Protocol. Questionnaires are filled in anonymously by the paramedic staff, and the analysis of the app use is anonymous. All collected data are only accessible for the research team. The research team is aware of data privacy regulations and committed to data protection. Data and information is not shared with other authorities or third parties. All collected data will be permanently destroyed or deleted 10 years after the study.

#### Sample size estimation

The sample size can only be estimated with a high degree of uncertainty because no data are available on language barriers on rescue operations. This underlines the ground-breaking character of the study. Moreover, no calculations are currently available on how paramedics perceive mutual understanding with foreign-language patients. This almost precludes estimating possible treatment effects. Because of the minimal risks to patient safety when the app is used in comparison to randomised controlled trials on drugs and biologics, we aim to include a sufficient number of patients to perform all necessary basal statistical analyses. “Sufficient” here means that even small effect sizes can be detected. Here, 50 to 75 patients in each interventional and control group 1 is described as appropriate [26]. In order to differentiate between the four recruitment sites, a total number of 200 to 300 included patients seems appropriate.

Braunschweig recorded 108 rescue operations per 1000 inhabitants per year and slightly more in an initial reception facility for refugees [15]. The districts of Helmstedt and Wolfenbüttel, where we assume that most of the emergency missions are carried out, are home to 6000 to 7000 foreigners [27]. If 20% of them have only limited German language proficiency [10], 1200 persons could be potential interventional persons. Considering Günther et al. 2016, 130 cases with non-German speaking patients are likely to occur within 1 year. When the crossing motorway corridors and non-German-speaking patients in the surrounding municipalities outside the district are taken into account, it appears possible to recruit an overall number of 400 to 600 patients with language barriers in 1 year.

We expect to receive all DIVI-Protocols of all rescue missions, including those with German-speaking patients for control group 2. This group includes in total 1600 patients at Wendhausen [28], 3500 for Königslutter [29], 6200 for Helmstedt [30], and 3500 for Braunschweig [31], based on the frequencies of served emergency cases in 2018.

#### Data analysis of the clinical trial

Characteristics of intervention and the two control groups are analysed using descriptive statistics regarding their demografics, medical conditions, assessed communication and native languages (where applicable), and aspects of the emergency operation. Differences between patients of the intervention group and the control groups will be tested for statistical significance using Chi-squared or Mann-Whitney *U*-Tests where applicable. In addition, the groups will be compared according to the recruitment centres in which patients were recruited. Relations between medical aspects of the emergency operation or demographic aspects and the assessed mutual understanding will be analysed using correlation analyses. Changes between control group 2 and the intervention group as to the perceived quality of understanding and to the quantity and quality of obtained information of patients will be analysed using paramedics’ feedback on questionnaires and comparing the content of the free text fields in the DIVI-protocols. In order to enable a comparison between non-German-speaking and German-speaking patients, a control group matched by age, sex, medical condition, and emergency situation will be drawn from the very large group of the German-speaking patients. Then, for example, on-scene times, rates of intubations, and requests for on-scene emergency doctor support can be compared.

### Study site and study setting

**Fig. 2:**
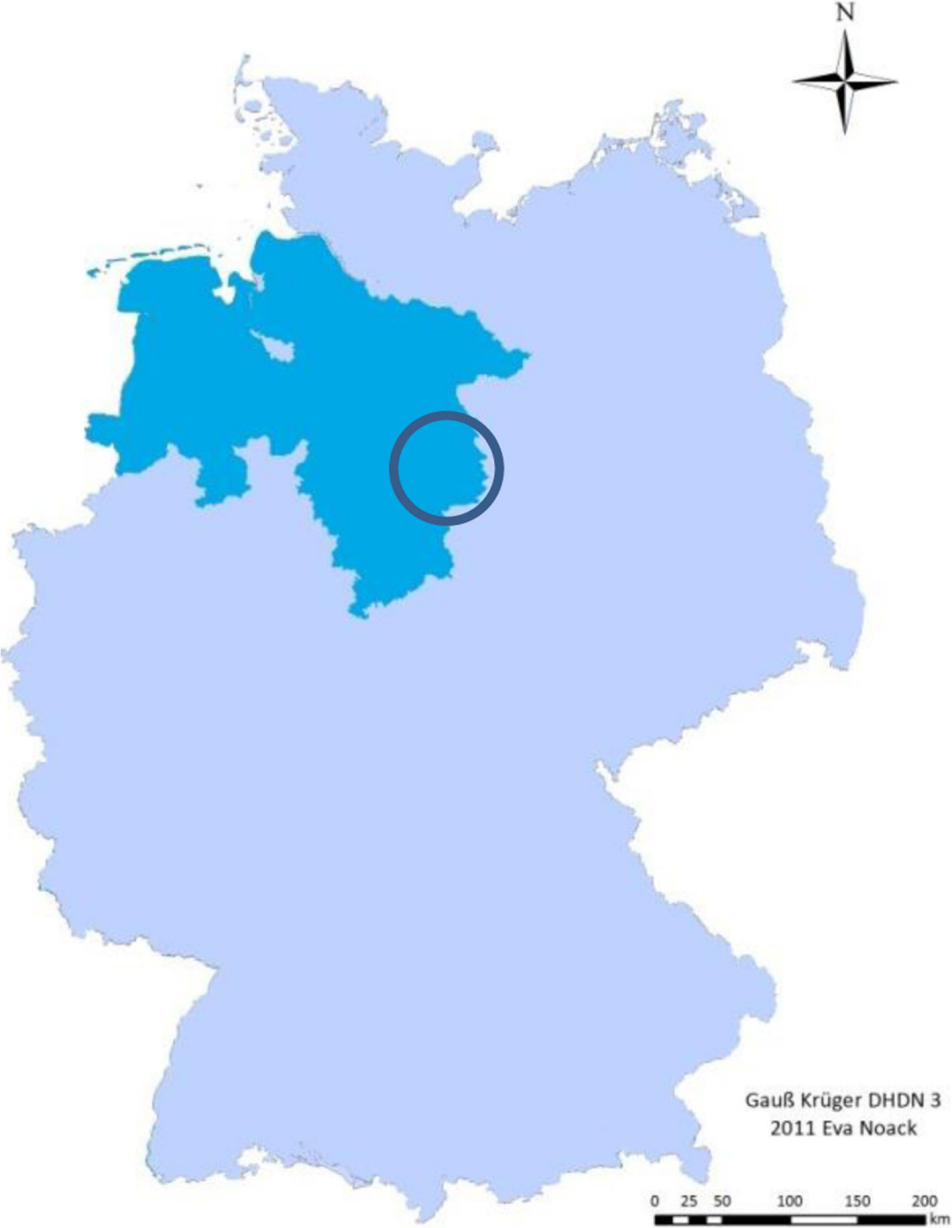
Location of the federal state Lower Saxony in Germany (cyan blue) and the study region (encircled in dark blue) (own map)

The study will take place at four emergency medical service stations in the east of Lower Saxony (cp. Fig. 2). Three of them, Königslutter, Wendhausen, and Braunschweig, are operated by the Order of Malta Ambulance Corps, the one in Helmstedt is operated by the district of Helmstedt. The emergency medical service stations Königslutter, Wendhausen, and Helmstedt are located in a rather structurally weak rural region adjoining the former inner-German border, whereas the station in Braunschweig is located in the second largest city in Lower Saxony.

Lower Saxony has 7.9 million inhabitants and is Germany’s second largest federal state by area. With 167 inhabitants per square kilometre, Lower Saxony is relatively sparsely populated compared to the average in Germany, which is 230 inhabitants per square kilometre, except for the urban sprawl around the capital Hanover and the adjacent city states Hamburg and Bremen [32].

The rescue services serve the five districts Helmstedt, Gifhorn, Wolfsburg, Braunschweig, and Wolfenbüttel. In the catchment area of the rescue services, 10% of the population have a foreign citizenship. This percentage varies between 6.1% in Wolfenbüttel and 18.0% in the city of Salzgitter (own calculations) [33–35]. The 15 main countries of origin of registered foreigners within the serviced area of the rescue services involved in this study are – in decreasing order – Turkey, Syria, Poland, Italy, Rumania, China, Iraq, Kosovo, Russia, Serbia (without Kosovo), Bulgaria, Greece, Afghanistan, Spain, and Croatia [33]. Additionally, one-fifth of agricultural seasonal workers in Germany work in Lower Saxony [36], and 1.6 million foreign tourists travelled to Lower Saxony in 2017 [6].

The rescue service stations Wendhausen and Helmstedt are located close to the motorway A2, which is the most important east-west route in northern Germany, connecting The Netherlands with its significant harbours with Poland and other countries in the region of north-eastern Europe. Therefore, a relatively high number of sick or injured transit passengers, especially tourists and truck drivers are patients of these rescue stations [28, 30].. The rescue station in Braunschweig serves as a central reception centre of refugees and asylum seekers (the Zentrale Anlaufstelle für Asylbewerber (ZASt)). The rescue stations in Wendhausen, Königlutter, and Helmstedt serve in structurally weak and rural areas where the next emergency hospital care is often a substantially long distance away. The location close to a frequented motorway means that the problem of providing paramedic care for non-German-speaking patients appears to be much higher than in other comparable rural regions.

### The communication tool

#### Development and content of the application software

The communication tool, which will be implemented and evaluated in this study, will be developed by the Department of General Practice at the University Medical Center of Göttingen and aidminutes GmbH, running as an application software (app). The researchers and developers have different professional backgrounds: they are medical doctors, emergency medical assistants, software engineers, designers, and cultural scientists. A team of physicians and paramedics of the University Medical Center of Göttingen will develop the content and structure, including the conceptual design, based on literature of emergency medicine, our own experience, and actively involving the paramedic staff of the participating rescue stations. Additionally, medical professionals other than the research team will review questions as to their accuracy, the validity of the answer considering the wording of the questions, and to check for completeness. Technical conception and realisation will be provided by software engineers and designers of aidminutes GmbH, who are experienced in app design and development.

The app will not support speech recognition because it does not yet work properly with most languages, especially when certain dialects are taken into account. Therefore, questions that paramedics usually ask patients without language barriers such as “What has happened?” or “Why did you call us?” cannot be transferred to the software because the foreign-language patient’s answer would not be understood. One challenge is therefore to pose questions in a way that the answer given by the patients is understandable despite the language barrier, which basically only allows questions that can be answered by “yes” or “no” or the request to point e.g. aching parts of the body.

Next to questions, the application software will include instructions and information that can be given to the patients, e.g. on medical or therapeutic procedures, so that patients can be guided through the rescue operation and physical examination.

Patients may be illiterate or might not be able to read in the emergency situation, therefore the app will primarily be audio-based. Professional interpreters experienced in translating medical information will translate the contents and narrate the translated questions. It is planned that the app will support the languages and dialects listed in Table 2.

The key element of the application software will thus be a module for rapid medical history taking to allow initial assessment, including important information that can be given to patients.

There will be a mode for adult patients and a mode for children patients, not only because the way to address patients and the wording of the questions differ, but especially because incidences of diseases, clinical pictures, and reasons for the paramedic encounter differ substantially between adults and children. Both modes will include the possibility to address questions and give information to third parties present, i.e. to patients’ relatives, children’s parents, or other accompanying adult persons. This can be of great importance, if patients themselves are not able to speak, e.g. due to a medical condition, or if a child patient cannot provide specific information.

Another great challenge is to group all questions and phrases in a way they can be easily found under time pressure. This implies that the content needs to be clustered according to the logic of rescue mission and resultant procedures, i.e. the staff’s way of thinking and acting, and to the probability that the questions are to be asked or the instructions are to be given in a certain situation during a rescue mission. This is where the action-oriented research approach will again be of great use. The grouping of the content is discussed in focus group discussions with the paramedic staff in the participating rescue stations. To observe whether the content can easily be found, the grouping will additionally be tested in practice relevant case studies. We will develop case trainings based on Löwe and Jahn 2017 [37].

#### Hardware of the communication tool

Because of the special circumstances of rescue missions, the use of different hardware on which the app is to be run (different smartphone and tablet models) and necessary accessories will be discussed and tested with the staff of the participating emergency rescue stations.

#### Integration of the communication tool into the rescue chain

**Fig. 3:**
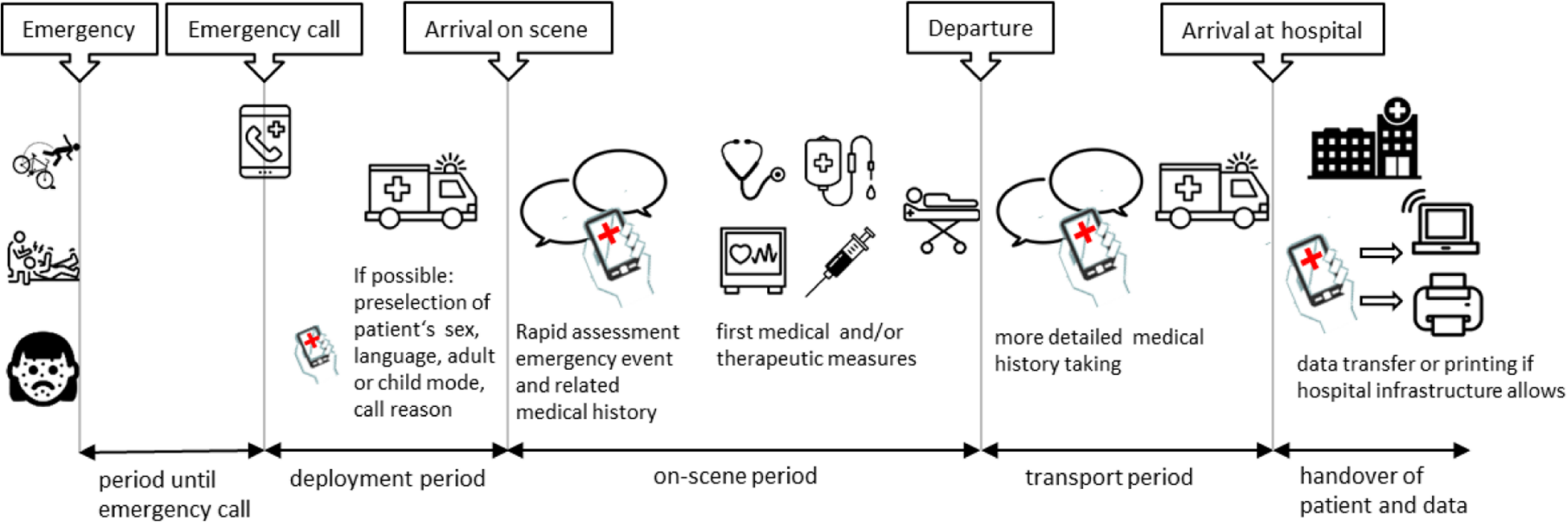
Integration of the communication tool into the rescue chain

When the notification of the emergency call indicates language barriers, paramedics may preselect the mode (adult or child), patient’s sex, and mother language on their way to the emergency site. On-scene, paramedics can perform a rapid assessment of patients’ complaints and pre-existing conditions – also with third parties – in order to decide which actions to take, e.g. whether an emergency doctor should be called to the scene, to which hospital the patient should be taken, and with which symptoms or suspected diagnosis the hospital should be notified. Paramedics may document the patient’s answers to the questions posed in the app. They may also explain therapeutic measures. A more detailed medical history can be taken during transport. This additional assessment may contain information on medication or known allergies and other information that might not be directly relevant for paramedic emergency treatment and transport but might be of interest for further treatment in the hospital. The app generates a report in German, which also serves as a documentation of the communication process. If possible, information can be shared with the hospital before or directly after arrival (cp. Fig. 3).

## Discussion

While there have been a quite a number of studies on the medical care of refugees and migrants in recent years, the emergency service with foreign-language patients has not yet been the subject of scientific concern. However, there are indications that language barriers pose a particular challenge to rescue workers and might endanger patients [16, 17].

For the first time, this study attempts to generate knowledge about language barriers in medical emergency operations. Furthermore, we will develop and implement the digital communication tool together with the end-users in an action-oriented research approach with an interdisciplinary team. This implies that the paramedic staff of four participating emergency medical service stations is involved in the whole development and implementation process. This certainly complex approach ensures that the app and the hardware meet the needs of the end-users and increases the likelihood of long-term implementation even after the study is completed. The use of technical solutions to overcome language barrier seems logical: professional interpreters, which are gold standard, can by nature not be present at the emergency scene. Lay interpreters, who are often family members, are problematic both in terms of translation accuracy and confidentiality [38, 39].

The app is expected to facilitate communication with foreign-language patients in paramedic care. A comparison of data on non-German-speaking patients with and without the developed communication tool is intended to show whether the app improves the perceived quality of understanding (primary outcome) and the quality and quantity of information received from the patient (secondary outcome). We expect that paramedics who use the app will be able to obtain information about their patients that they could not collect otherwise. This may lead to a better and safer provision of paramedic care.

## Supplementary information

**Additional file 1.** Questionnaire to assess the experience of paramedics on rescue missions with non-German-speaking patients (translated from German).

## Data Availability

The datasets used and analysed during the current study are available upon reasonable request, under consideration the existing ethics committee vote and the legal framework conditions (Emergency Services Law of Lower Saxony) from the authors.

## Abbreviations

app: application software
COPD: Chronic obstructive pulmonary disease
DIVI: Deutsche Interdisziplinäre Vereinigung für Intensiv- und Notfallmedizin e.V., i.e. German Interdisciplinary Association for Intensive and Emergency Medicine
NACA: National Advisory Committee for Aeronautics
NEF: Notarzteinsatzfahrzeug, i.e. non-transporting emergency physician rapid response car
NotSanG: Notfallsanitätergesetz. i.e. Emergency Paramedics Act
RTW: Rettungstransportwagen, i.e. patient-transporting ambulance
SIDS: Sudden infant death syndrome
STEMI: ST-elevation myocardial infarction
SUS: System Usability Scale
