# Supplementary material for: Overcoming language barriers in paramedic care: a study protocol of the interventional trial ‘DICTUM rescue’ evaluating an app designed to improve communication between paramedics and foreign-language patients
Source: BMC Health Serv Res. 2020 Mar 18;20:223. doi: 10.1186/s12913-020-05098-5 (PMC7079507; doi:10.1186/s12913-020-05098-5)
Supplement: Supplementary file 1 — Additional file 1. Questionnaire to assess the experience of paramedics on rescue missions with non-German-speaking patients (translated from German). [file 12913_2020_5098_MOESM1_ESM.docx]

| **Questionnaire on emergencies with non-German-speaking patients**  My opinion on the rescue mission:  I perceived a language barrier with the patient 🞏 yes 🞏 partly 🞏 no  The patient probably spoke best (mother tongue): _______________________  I communicated with the patient…  🞏 in German  🞏 with hands and feet  🞏 using Google translator or similar  🞏 someone else has translated, namely _______________________  🞏 in another language, namely _______________________  🞏 other _______________________  I felt the communication with the patient as a whole was…  very easy 🞏 🞏 🞏 🞏 🞏 very difficult  I have received …  all 🞏 🞏 🞏 🞏 🞏 not any  …necessary information from the patient  I would have liked to obtain this information from the patient: _______________________  I could tell/explain …  everything 🞏 🞏 🞏 🞏 🞏 nothing  … to the patient  I would have liked to share this information with the patient: _______________________ |
| --- |
